# Repair Bond Strength of Milled and 3D‐Printed CAD/CAM Hybrid Materials Using Different Repair Protocols: An In Vitro Study

**DOI:** 10.1002/cre2.70380

**Published:** 2026-05-20

**Authors:** Marko Milicevic, Florian Beuer, Nico Henning, Jamila Yassine, Franziska Schmidt, Jonas Rechlin, Elisabeth Prause

**Affiliations:** ^1^ Department of Prosthodontics, Geriatric Dentistry and Craniomandibular Disorders Charité‐Universitätsmedizin Berlin, corporate member of Freie Universität Berlin and Humboldt‐Universität zu Berlin Berlin Germany

**Keywords:** 3D‐printing, CAD/CAM, dentistry, prosthodontics, repair protocol, shear bond strength

## Abstract

**Objectives:**

The aim of this study was to evaluate the bond strength of different computer‐aided design/computer‐aided manufacturing (CAD/CAM) hybrid materials for milling and 3D‐printing with and without thermocycling. Different repair protocols were tested.

**Materials and Methods:**

Two milled and one experimental 3D‐printed hybrid material were tested with three repair systems (MO [universal primer and dental adhesive]; GC [Repair Kit]; Voco Cimara [Repair Kit]). A total of 180 specimens were prepared (*n* = 60 per material). Half underwent thermocycling (30,000 cycles, 5°C–55°C), while the others were stored in distilled water (37°C). Shear bond strength was determined using a universal testing machine. Fracture modes were analyzed microscopically, and selected samples were examined via scanning electron microscopy. Data were analyzed by three‐way ANOVA followed by Tukey's and Bonferroni post hoc tests (*p* < 0.05).

**Results:**

The results of the present study showed that the choice of repair protocol and the tested material significantly influenced the shear bond strength. MO achieved the highest shear bond strength values among all tested materials before and after thermocycling. After thermocycling, only GC in combination with the experimental 3D‐printed material depicted an increase in shear bond strength (12.55 → 14.51 MPa). Regarding the tested milled materials, thermocycling reduced shear bond strength across all protocols.

**Conclusions:**

Shear bond strength was significantly influenced by the type of CAD/CAM material and repair protocol used. Thermocycling reduced shear bond strength in milled materials, while the 3D‐printed material showed improved values with specific protocols. Clinicians should be aware that both the choice of CAD/CAM material and the repair protocol significantly affect bond strength.

## 1 Introduction

In recent years, computer‐aided design (CAD) and computer‐aided manufacturing (CAM) have become an important process in dentistry (Shin et al. 2020), and it contributed a lot to the development of new clinical workflows (Espinar et al. 2023). Especially, subtractive manufacturing (milling) using composites has been well‐adapted to manufacturing processes (Oudkerk et al. 2024). It was confirmed that milled restorations show high accuracy regarding the anatomic form, the occlusal and interproximal contacts (Zaghloul et al. 2014; Akbar et al. 2006; Reiss 2003; Peumans et al. 2007) and the possibility to repair them intraorally (Oudkerk et al. 2024; Zaghloul et al. 2014; Mainjot et al. 2016).

In addition to the further development of CAD/CAM, additive manufacturing (3D printing) has gained in popularity (Shin et al. 2020; Stansbury and Idacavage 2016) due to a more economical material consumption and the possibility to print multiple restorations at the same time (Espinar et al. 2023; Stansbury and Idacavage 2016; Della Bona et al. 2021).

Still, the most commonly available CAD/CAM restorative materials are ceramics and composites (Elsaka 2014; Coldea et al. 2013). However, newly available CAD/CAM hybrid materials for subtractive and additive manufacturing appeared on the dental market. These are ceramic‐reinforced polymers, combining the positive aspects of both material groups (Duarte et al. 2016). They are mainly used for single tooth restorations such as inlays, onlays, veneers, and crowns (Duarte et al. 2016).

Clinical failures regarding single tooth restorations were mostly described as chippings, fractures, or secondary caries (Duarte et al. 2016; Nguyen et al. 2014, 2013). Furthermore, intraceramic defects, trauma, and parafunctional habits were often mentioned to cause fractures of all‐ceramic restorations (Elsaka 2015; Ozcan and Niedermeier 2002). In the case of chippings and fractures, a complete removal of the restoration with subsequent renewal became necessary. Additional loss of tooth structure due to another preparation makes this procedure extremely invasive (Duarte et al. 2016; Nguyen et al. 2012). Besides, additional costs for the replacement became necessary, and further trauma to the tooth could occur (Elsaka 2015; Hickel et al. 2013; Kim et al. 2005).

To avoid an invasive removal of a fractured restoration, advances in adhesive dentistry led to intraoral repair possibilities. Here, a resin‐bonded composite technique presented a cheaper, functional, and esthetically pleasing treatment alternative to a complete restoration replacement (Duarte et al. 2016; He and Swain 2011; Kassem et al. 2012).

Since then, different protocols for an intraoral repair have been described (Zaghloul et al. 2014). All these protocols promoted a surface treatment of the repaired substrate to enhance the adhesive bonding capacity to the repairing composite material (Zaghloul et al. 2014; Blum et al. 2012; Lucena‐Martín et al. 2001; Özcan et al. 2009; Brentel et al. 2007; Özcan 2002; Kukiattrakoon and Thammasitboon 2012). Other studies mostly intended to mechanical, chemical, or physico‐mechanical pretreatment of the repairing surfaces (Zaghloul et al. 2014). Mechanical pretreatment was conducted using airbone particle abrasion with alumina oxide particles (Blum et al. 2012; Lucena‐Martín et al. 2001; Özcan et al. 2009). Chemical pretreatment used phosphoric or hydrofluoric acid for conditioning the repairing surfaces (Zaghloul et al. 2014; Lucena‐Martín et al. 2001; Brentel et al. 2007). Physico‐mechanical pretreatment was achieved with the help of the tribosilica coating technique (Zaghloul et al. 2014; Özcan 2002).

In order to simplify the clinical steps, repair protocols for CAD/CAM hybrid materials have been developed (Elsaka 2015). Therefore, the present study intends to evaluate different repair protocols at CAD/CAM hybrid materials for milling and printing with and without thermocycling. So far, no data are available regarding CAD/CAM hybrid materials for 3D printing. Therefore, this study aimed to compare the bond strength of printed and milled CAD/CAM hybrid materials repaired with different adhesive systems, before and after thermocycling. Fracture modes will be analyzed using scanning electron microscopy (SEM). To test the null hypothesis that no significant differences in the bond strength between the repair protocols and materials occur, the following in vitro study was conducted.

## 2 Materials and Methods

Three different CAD/CAM hybrid materials for subtractive and additive manufacturing were tested in the present study. Regarding repair protocols, a ceramic resin composite was used (Table 1).

**Table 1:** Analyzed materials.

Material	Composition	Manufacturer	Manufacturing methods	Code
Experimental material	Ceramic‐filled (30–50 wt% inorganic fillers; particle size 0.7 µm) silanized dental glass, methyl benzoylfor‐mate, diphenyl (2, 4, 6‐trimethylbenzoyl) phosphine oxide hybrid material	/	Additive manufacturing	3D
Vita Enamic	Polymer infiltrated (Urethane dimethacrylate, Triethylenglycoldimethacrylat 14 wt%) feldspar ceramic network (86 wt%)	VITA Zahnfabrik, Bad Sackingen, Germany	Subtractive manufacturing	VE
Voco Grandio	Resin nanohybrid composite (86 wt% inorganic fillers; particle size 20–60 nm), embedded in a polymer matrix (14% UDMA + DMA)	Voco, Cuxhaven, Germany	Subtractive manufacturing	VG
Ceram.x spectra universal flow	Universal nano ceramic resin composite (glass filler content 76 wt%; particle size 10 nm), methacrylate modified	Dentsply Sirona, Charlotte, USA	Direct composite material	CX

Rectangular specimens of each of the CAD/CAM millable and printable hybrid materials were created using an STL dataset designed in FreeCAD (Version 0.19.2 for Windows, FreeCAD Software Foundation) (Gayer et al. 2016). The dimensions of the samples were 13 × 10 × 2 mm.

Samples made of an experimental CAD/CAM hybrid material for additive manufacturing (3D) were printed using digital light processing (DLP) technology. Printing was performed at 90° to the build platform with a layer thickness of 50 μm. After removal of support structures, the samples underwent a two‐step cleaning process. Initially, they were sonicated in 96% ethanol for 3 min, followed by rinsing with fresh ethanol and drying with oil‐free compressed air. The samples were then sandblasted at a pressure of 1.5 bar to remove debonded ceramic particles for 10 s (Perlablast micro, 50 µm, BEGO, Bremen, Germany). Final curing was performed in a light‐curing unit (Otoflash, BEGO) using 1500 flashes per side for 3 min.

Regarding Vita Enamic (Vita Zahnfabrik, Bad Säckingen, Germany) (VE) and Voco Grandio (Voco, Cuxhaven, Germany) (VG), samples were manufactured from CAD/CAM blocks using a 5‐axis milling machine (vhf K5, Ammerbuch, Germany). The bonding surface of each specimen was ground and polished by using 320‐, 500‐ 800‐, and 1200‐grit silicon carbide papers (Hermes Schleifmittel, Hamburg, Germany) under water cooling for 30 s each. Subsequently, the specimens were cleaned in an ultrasonic bath with distilled water for 5 min and dried with oil‐free compressed air.

For each material, 60 specimens were manufactured, resulting in a total of 180 samples (*n* = 180). An a priori sample size calculation was performed based on previously published shear bond strength (SBS) data of CAD/CAM materials (Kanat et al. 2014; Mangoush et al. 2021). Assuming a medium effect size (*f* = 0.25), a significance level of *α* = 0.05, and a statistical power of 80% (1−*β* = 0.80), a minimum sample size of *n* = 10 per subgroup was determined to be sufficient for three‐way ANOVA. The primary endpoint of this study was SBS, expressed in megapascals (MPa).

**Figure 1:**
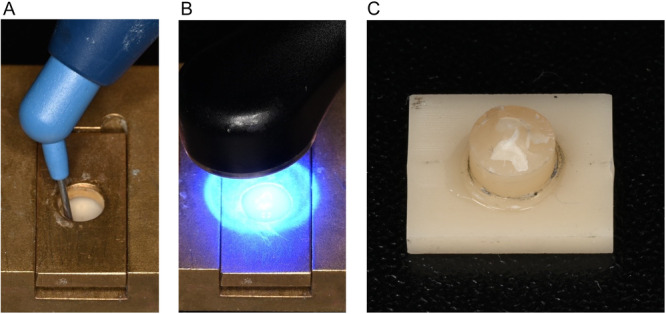
Preparation of specimens: (A) A cylindrical mold was used to apply the direct composite material; (B) light curing was performed for polymerization of the cylinder; (C) prepared specimens consisting of the tested material and the cylindrical test body, which was applied using different repair protocols.

A transparent cylindrical mold with a diameter of 5 mm and 2.5 mm in height was produced to apply the direct composite material. The nanoceramic composite (Ceram.x spectra universal flow, Dentsply Sirona, Charlotte, USA) (CX) for direct restorations was manually layered and light cured for 40 s (VALO Cordless, Ultradent Products, Cologne, Germany) from each side. After curing, the ceramic‐resin composite block was removed from the mold and was again exposed to 40 s of light curing (Figure 1A–C).

Half of the specimens of each tested material group and repair protocol (*n* = 10) were selected for artificial aging. According to the International Organization for Standardization (ISO) standard 10477 half of the samples of each material group and repair protocol were stored in 37°C distilled water for 24 h, while the remaining samples were subjected to 30,000 thermal cycles in 5°C and 55°C water baths with 30 s of dwell time in each water bath and 5 s of transfer time (Mao et al. 2024).

The following three repair protocols were applied in the present study.

Group 1 (MO): Monobond plus (Ivoclar, Schaan, Liechtenstein) combined with Optibond FL adhesive (Kerr Dental, Orange, CA, USA). Monobond plus was applied for 60 s and removed using oil‐free air. Subsequently, Optibond FL was applied, gently worked in for 15 s, air‐thinned for 3 s, and light‐cured for 20 s.

Group 2 (GC): GC Repair Kit (GC Corporation, Tokyo, Japan), consisting of the G‐Multi Primer and the G‐Premio Bond. The primer was applied and air‐thinned, followed by the application of the bonding material, air‐thinned for 5 s, and light‐cured for 10 s.

Group 3 (VC): Voco Cimara (Voco), consisting of the silane and adhesive. After additional mechanical pretreatment, the silane was applied and left to dry in the air for 2 min. Afterwards, the adhesive was applied, air‐thinning for 5 s, and light‐curing for 20 s.

SBS was measured using a universal testing machine (RetroLine Z010, Zwick/Roell, Ulm, Germany) according to DIN EN ISO 10477 (Mao et al. 2024). Loading was applied parallel to the bonding surface at a cross‐head speed of 0.5 mm/min until material fracture occurred. The failure load (*N*) was recorded, and SBS values were converted into megapascals (MPa) by dividing the fracture load (*F*) in Newton by the bonding surface area (*A*) in mm^2^:

$$\mathrm{SBS}=\frac{F}{A}=\left[\frac{N}{{\mathrm{mm}}^{2}}\right]$$

The calculation of the surface area of the samples is standardized based on the mold dimensions during sample production:

$$A=d^{2}\;x\;\frac{\pi }{4}$$

The adhesive surface area, with a diameter of 5 mm, is approximately 19.63 mm^2^.

Following debonding, the fractured interfaces were analyzed using a digital microscope (Keyence VHX‐500, Frankfurt, Germany) at 30× magnification to assess the failure mode. The failure modes were classified as follows (Günal‐Abduljalil et al. 2021): (1) Adhesive failure occurring at the bonding interface; (2) cohesive failure within the adhesive luting material or the composite resin itself; and (3) mixed failure, characterized by a combination of adhesive and cohesive failures. Additionally, one randomly selected specimen from each group was examined using SEM at 1000× magnification to evaluate the topography of the fractured surface.

A Phenom ProX scanning electron microscope (PhenomWorld, Eindhoven, Netherlands) was used for the SEM examinations. Imaging was performed using a backscattered electron detector (BSD). The samples were examined at a vacuum pressure of 60 Pa and an acceleration voltage of 15 kV. Two images of each sample were taken at 1000× and 3000× magnification.

To perform statistical analyses, IBM SPSS Statistics 29.0 (IBM Corp., Armonk, NY, USA) was used. Descriptive statistics were carried out, and boxplots were used to visualize the distribution of SBS (MPa) across groups. Normality of the residuals was assessed using the Shapiro–Wilk test and visual inspection of Q–Q plots. The assumption of homogeneity of variances was verified using Levene's test. Independence of observations was ensured through the experimental design. The influence of the test material, adhesive system, and TC on SBS was analyzed using a three‐way analysis of variance (3‐way ANOVA), including all two‐way and three‐way interaction terms. Post‐hoc comparisons were conducted using Tukey's and Bonferroni correction methods. The frequencies of failure modes (adhesive, cohesive, mixed) were illustrated using bar charts. Statistical significance was set at *p* < 0.05. Specimens for which fracture surface classification was not possible (e.g., due to damage during post‐test handling) were excluded from the failure‐mode analysis; the effective sample size for the fracture‐mode evaluation may therefore be smaller than the number of specimens used in the SBS analysis.

## 3 Results

Out of the 180 specimens prepared, 174 were successfully analyzed. Six specimens, all fabricated from VG and subjected to TC, were excluded due to premature adhesive failure before SBS testing. Among these, five specimens were repaired using the GC, and one with VC.

Overall, the SBS values were significantly lower following TC across almost all tested materials (*p* < 0.001). Without TC, all substrates demonstrated significantly higher SBS. This effect was particularly pronounced in VG, where a marked decline in SBS after TC corresponded with the six instances of early adhesive failure (Material × TC interaction: *p* < 0.001); 3D exhibited the highest overall SBS values, especially in the absence of TC (main effect of material: *p* < 0.001). After TC, SBS values showed reduced variability, suggesting a stabilizing yet bond‐reducing effect of the aging process.

With one notable exception, SBS values decreased after TC across all materials and adhesive combinations (Table 2). This reduction was statistically significant for all VG and VE subgroups (*p* < 0.001), while the changes observed in the 3D subgroups did not reach significance. Only the combination of 3D with GC exhibited an increase in SBS after TC (12.55 → 14.51 MPa), highlighting a unique interaction between this adhesive and substrate (Adhesive × TC interaction: *p* = 0.049).

**Table 2:** SBS‐values before and after TC for the tested materials.

Material	Repair protocol	Average value without TC (MPa)	Standard deviation (SD)	Average value with TC (MPa)	Standard deviation (SD)
3D	VC	28.19	7.28	20.93	10.11
	GC	12.55	4.72	14.51	8.15
	MO	26.06	5.91	19.01	8.57
VG	VC	18.81	3.57	6.23	3.38
	GC	15.23	2.68	2.82	2.56
	MO	27.15	6.14	10.34	3.82
VE	VC	22.52	5.11	8.36	2.38
	GC	15.22	3.46	5.04	1.75
	MO	26.92	3.88	13.05	4.05

Regarding 3D, in 57 out of 60 samples, failure occurred at least partially within the substrate rather than purely at the adhesive interface, indicating that the SBS exceeded the inherent structural stability of the material. Additionally, six VG specimens experienced complete adhesive failure. VE showed no material fractures.

**Figure 2:**
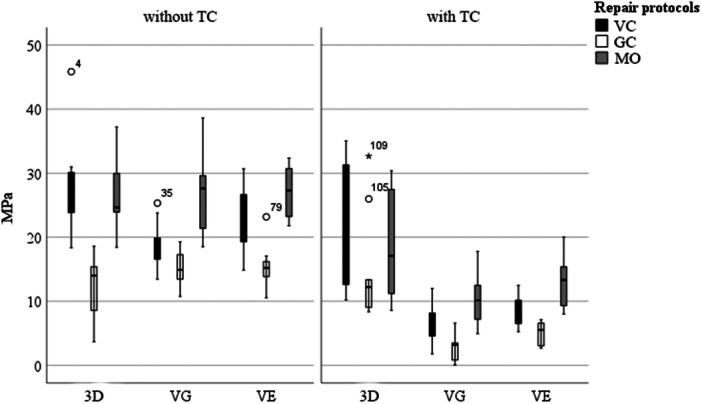
Evaluation of the tested materials (experimental printed material, 3D; Voco Grandio, VG, and Vita Enamic, VE) before and after TC with respect to the applied repair protocol.

Concerning the repair protocols, GC consistently resulted in the lowest SBS values across most materials (main effect of adhesive system: *p* < 0.001), while MO performed better than GC but remained below VC. The highest SBS values overall were achieved with 3D repaired using VC, decreasing from 28.19 MPa before TC to 20.93 MPa afterwards (Figure 2 and Table 2). Conversely, the lowest SBS after TC was recorded for VG repaired with GC, with a decrease from 15.23 to 2.82 MPa (*p* < 0.001) (Figure 2 and Table 2). Boxplots further revealed greater variability in SBS without TC, while post‐TC results demonstrated reduced dispersion (Figure 2).

Regarding specimens included in statistical analyses, all 3D samples were available (*n* = 20 per adhesive). For VG, VC had 19 specimens (95.0%), GC 15 specimens (75.0%), and MO again 20 specimens (100.0%). For VE, all adhesive systems showed 100% inclusion (*n* = 20). 3D displayed a tendency for substrate failure while maintaining intact adhesive bonds, suggesting its potential as a robust repair substrate due to its relatively high SBS retention after TC. The singular increase in SBS observed for 3D repaired with GC after TC warrants further investigation.

Shear bond strength testing was performed in accordance with DIN EN ISO 10477:2020. The maximum load at failure was recorded in Newtons and converted into shear bond strength values in MPa by dividing the failure load by the bonded surface area. After debonding, the fracture surfaces of all specimens were visually examined and documented as supplementary findings. Failure modes were classified according to predefined criteria as adhesive failure, cohesive failure, mixed adhesive–cohesive failure, or excluded. Adhesive failure was defined as separation predominantly at the interface between the substrate and the repair material. Cohesive failure was defined as fracture predominantly within one of the bonded materials. Mixed failure was defined as a combination of adhesive and cohesive fracture characteristics. Specimens were excluded from failure‐mode analysis when fracture patterns could not be reliably assigned or when pre‐test failure/technical artefacts occurred. Failure modes were evaluated descriptively and additionally stratified according to thermocycling status (specimens with and without TC).

Without TC, mixed adhesive–cohesive failures were the most frequent across all adhesives, especially in combination with 3D. Cohesive failures were also notably common, indicating strong bonding performance, while adhesive failures were relatively rare (Table 3).

**Table 3:** Failure rates (%) of each tested material with respect to the repair protocol applied.

Material		Without TC (%)			With TC (%)		
		Adhesive failure	Adhesive/Cohesive failure	Cohesive failures	Adhesive failure	Adhesive/Cohesive failure	Cohesive failures
3D	VC		20	80	10	80	10
	GC	10	60	30	10	30	60
	MO			100	14.3	14.3	71.4
VG	VC	100			80	20	
	GC	10	90		100	0	
	MO		10	90	10	90	
VE	VC		70	30	20	80	
	GC	50	50		100		
	MO		10	90		30	70

**Figure 3:**
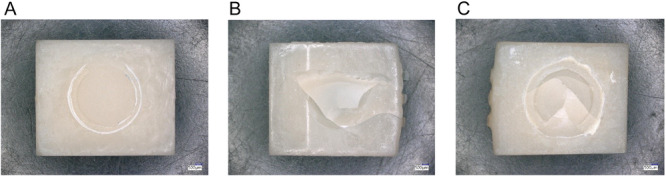
Different failure modes: (A) Adhesive failure of VG repaired with VC; (B) cohesive failure of 3D repaired with MO; (C) mixed adhesive–cohesive failure of 3D repaired with MO. The analysis of the fracture mode was conducted in accordance with the findings of Mao et al. (2024).

**Figure 4:**
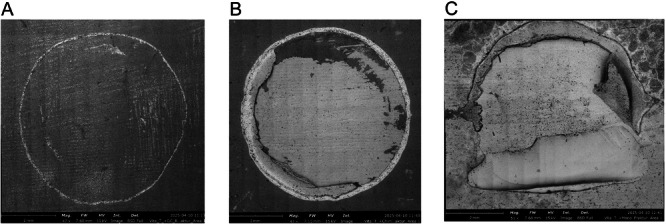
SEM images of different failure modes after TC: (A) Adhesive failure of VE repaired with GC, magnification 47×; (B) cohesive failure of VE repaired with VC, magnification 47×; (C) mixed adhesive–cohesive failure of VE repaired with MO, magnification 51×.

After TC, a shift in failure modes was observed. Adhesive failures became more prevalent, particularly in specimens repaired with GC (Figure 3A and Figure 4A). The fracture surface exhibits a smooth and relatively featureless morphology, indicating complete interfacial separation between the adhesive and the substrate. No significant adhesive residues remain, suggesting poor adhesion at the interface. Cohesive failures occurred mainly for VE (Figure 3B and Figure 4B), while mixed‐mode failures remained common in 3D specimens (Figure 3C and Figure 4C).

This distribution supports the finding that TC compromises the adhesive interface, especially in less compatible material–adhesive combinations, while more stable pairings (e.g., 3D with VC or MO) continue to demonstrate predominantly cohesive or mixed failures even after aging.

## 4 Discussion

The aim of the present clinical study was to evaluate the SBS of different CAD/CAM hybrid materials for additive and subtractive manufacturing using different repair protocols. The null hypothesis that there was no difference regarding the bond strength of the tested materials after different repairing procedures and thermocycling had to be rejected.

Depending on the extent and cause of the ceramic restoration fracture, intraoral repair with resin composite may be considered a simple alternative to extraoral repair (Elsaka 2015; Ozcan and Niedermeier 2002; Goia et al. 2006; Özcan and Vallittu 2003; Kanat et al. 2014). Although several repair protocols for all‐ceramic materials, based on different conditioning protocols, are available on the dental market, it remains challenging for clinicians to choose the most suitable system that will ensure reliable results (Elsaka 2015; Blum et al. 2012; Özcan et al. 2009). In recent years, CAD/CAM hybrid materials, such as polymer‐infiltrated ceramics or resin‐matrix materials with a high ceramic content, have been developed. These materials aim to combine the advantageous mechanical strength of ceramics with the flexibility of resin (Mao et al. 2024; Mangoush et al. 2021; Sripetchdanond and Leevailoj 2014). By replacing the glass matrix of conventional ceramics with a polymer matrix, CAD/CAM hybrid materials are more resistant to cracks and exhibit mechanical properties similar to enamel and dentin. As a result, they are closer to natural materials and less abrasive to them (Mao et al. 2024; Silva et al. 2023; Venturini et al. 2019). Moreover, with the ongoing advancements in light‐curing and 3D‐printing technologies, it is now possible to print CAD/CAM hybrid materials for single‐tooth restorations (Mao et al. 2024; Grzebieluch et al. 2021).

So far, CAD/CAM hybrid materials might be more repairable than all‐ceramic materials due to their amount of polymers. However, little scientific evidence exists on how to optimally repair such materials intraorally. Repair of ceramic restorations requires the surface to be treated in a way that enhances the adhesion of the repair resin composite to the hybrid materials' surface (Mao et al. 2024). In the present study, different surface pretreatments were conducted based on the recommendations of the manufacturers for the tested CAD/CAM hybrid materials for milling and printing. The present study aimed to determine the SBS of CAD/CAM hybrid materials for milling and printing using three intraoral repair protocols. The SBS test is one of the most commonly used methods to assess the bond strength of a material (Mao et al. 2024; Mangoush et al. 2021; Burke et al. 2008). It is also simple to perform and highly reproducible (Mao et al. 2024; Mangoush et al. 2021; Burke et al. 2008). Additionally, previous studies have shown that the roughness and wettability of CAD/CAM material surfaces can influence bond performance (Mao et al. 2024; Chuenjit et al. 2021; Porto et al. 2023).

Restorations commonly fail after being exposed to a humid oral environment. Aging conditions can cause alterations to the surfaces of restorative materials, and thus, the aging process of the restoration should be considered when planning its repair. SBS is therefore influenced by TC and mainly reduced after TC (Mehl et al. 2016; Pitta et al. 2020; Rues et al. 2017). In this study, specimens were aged using TC. Specimens were subjected to 30,000 thermal cycles in 5°C and 55°C water baths. TC led to a reduction in bond strength across all milled materials (VE and VG) and repair protocols, while the 3D‐printed material showed more stable SBS values that were not significantly affected by TC.

Across all groups, Monobond Plus and Optibond FL achieved the highest bond strengths both before and after TC. This system's superior performance can be attributed to the synergistic effect of silane coupling agents and phosphoric acid methacrylates, which chemically interact with both the ceramic and polymer components of hybrid materials. Optibond FL, as a three‐step etch‐and‐rinse adhesive, ensures deep resin infiltration and stable hybrid layer formation, leading to durable adhesion even after TC. In contrast, the simplified universal system (GC Repair Kit) demonstrated limited chemical interaction and micromechanical retention, particularly with the milled materials VE and VG, resulting in lower SBS values and more frequent adhesive failures.

For milled hybrids (VE, VG), TC significantly reduced SBS across all protocols, suggesting that their higher ceramic content and denser structure may increase susceptibility to interfacial stresses and hydrolytic degradation. The 3D‐printed material, in contrast, exhibited more cohesive failures and less reduction in SBS, indicating better internal stress distribution and improved repair potential. These findings underline that the material's polymer‐to‐ceramic ratio and network configuration are key determinants of long‐term repair durability.

The observed differences among protocols and materials highlight the need for material‐specific repair strategies. Clinicians should consider both the chemical composition of the hybrid material and the bonding mechanism of the repair system when performing intraoral repairs. Among the tested systems, MO provided the most reliable overall performance and may be recommended for hybrid CAD/CAM restorations under clinical conditions.

The present findings are in agreement with previous studies demonstrating that the bond strength of CAD/CAM materials is not only dependent on the repair protocol but also on the chemical composition of the material and the applied adhesive system. Additionally, factors such as different adhesive formulations, including universal adhesives, have been shown to significantly influence bond strength to enamel and restorative materials (Beltrami et al. 2016). Similarly, variations in photopolymerization protocols and photoinitiator systems may affect the degree of conversion and ultimately the mechanical performance of resin‐based materials (Delgado et al. 2019). These aspects should be considered in future studies to further optimize repair strategies for CAD/CAM hybrid materials.

Some limitations of the present study must be noted. Only three hybrid CAD/CAM materials and three repair systems were examined, and surface pretreatments followed manufacturer‐specific protocols, which may have influenced the outcomes. Printing was performed in a single (90°) orientation, intentionally representing a mechanically less favorable condition. Further research should investigate different printing orientations, additional surface conditioning strategies, and long‐term aging or fatigue testing to better simulate clinical environments. Complementary test methods, such as tensile or microtensile bond strength analysis, could also provide more detailed insights into the adhesive interface behavior. Furthermore, the lack of clinical trials limits the direct transferability of the present in vitro findings to clinical conditions.

## 5 Conclusions

The present study showed that both the repair protocol and the material used significantly affected the SBS. MO showed the highest SBS values before and after TC. 3D repaired with VC had a higher SBS value than MO.

Notably, only GC with 3D showed an increase in SBS after TC. In contrast, for subtractively manufactured materials like VE and VG, TC led to a decrease in SBS across all protocols. The study concludes that selecting the right repair protocol is crucial, especially with the availability of new CAD/CAM hybrid materials that facilitate intraoral repairs.

## Author Contributions

Conceptualization: Florian Beuer and Elisabeth Prause. Methodology: Nico Henning and Marko Milicevic. Investigation: Marko Milicevic. Formal analysis: Jamila Yassine, Franziska Schmidt, Jonas Rechlin, and Marko Milicevic. Writing – original draft: Elisabeth Prause. Writing – review and editing: Marko Milicevic, Florian Beuer, Jamila Yassine, Franziska Schmidt, Jonas Rechlin, and Elisabeth Prause. Supervision: Florian Beuer and Elisabeth Prause.

## Funding

The authors have nothing to report.

## Ethics Statement

The present clinical study was approved by the local Ethics Committee in 2020 (application number: EA1/250/23). Since then, it has been conducted in accordance with the Declaration of Helsinki on Ethical Principles for Medical Research.

## Consent

The authors have nothing to report.

## Conflicts of Interest

The authors declare no conflicts of interest.

## Data Availability

The datasets used and/or analyzed during the current study are available from the corresponding author on reasonable request.
